# T Oligo-Primed Polymerase Chain Reaction (TOP-PCR): A Robust Method for the Amplification of Minute DNA Fragments in Body Fluids

**DOI:** 10.1038/srep40767

**Published:** 2017-01-17

**Authors:** Yu-Shin Nai, Tzu-Han Chen, Yu-Feng Huang, Mohit K. Midha, Hsin-Chieh Shiau, Chen-Yang Shen, Chien-Jen Chen, Alice L. Yu, Kuo Ping Chiu

**Affiliations:** 1 Department of Biotechnology and Animal Science, National Ilan University, Yilan, Taiwan; 2 Genomics Research Center, Academia Sinica, Taipei, Taiwan; 3 Institute of Biochemistry and Molecular Biology, National Yang-Ming University, Taipei, Taiwan; 4 Institute of Biomedical Sciences, Academia Sinica, Taipei, Taiwan; 5 Institute of Stem Cell and Translational Cancer Research, Chang Gung Memorial Hospital at Linkou and Chang Gung University, No. 5, Fu-Shin St., Kuei Shang, Taoyuan 333, Taiwan; 6 Department of Pediatrics, University of California in San Diego, CA USA; 7 Department of Life Sciences, College of Life Sciences, National Taiwan University, Taipei, Taiwan

## Abstract

Body fluid DNA sequencing is a powerful noninvasive approach for the diagnosis of genetic defects, infectious agents and diseases. The success relies on the quantity and quality of the DNA samples. However, numerous clinical samples are either at low quantity or of poor quality due to various reasons. To overcome these problems, we have developed T oligo-primed polymerase chain reaction (TOP-PCR) for full-length nonselective amplification of minute quantity of DNA fragments. TOP-PCR adopts homogeneous “half adaptor” (HA), generated by annealing P oligo (carrying a phosphate group at the 5′ end) and T oligo (carrying a T-tail at the 3′ end), for efficient ligation to target DNA and subsequent PCR amplification primed by the T oligo alone. Using DNA samples from body fluids, we demonstrate that TOP-PCR recovers minute DNA fragments and maintains the DNA size profile, while enhancing the major molecular populations. Our results also showed that TOP-PCR is a superior method for detecting apoptosis and outperforms the method adopted by Illumina for DNA amplification.

Conventional Polymerase chain reaction (PCR) utilizes paired primers to define the boundaries of the target region and to prime DNA synthesis. Although PCR using paired-primers conveys evident advantages, the strategy has an intrinsic disadvantage as far as full-length amplification of linear DNA is concerned. Based on the approach, it is essential for the two termini of target DNA fragment to ligate to two different adaptors, so to permit the binding of two different PCR primers, each complementary to a specific site in the adaptor, for full-length amplification of the target DNA. Since every terminus has an equal chance to ligate to either adaptor, half of the DNA fragments are expected to ligate to only one type of adaptor, resulting in a 50% loss of the target sequence information. This strategy was carried over to SOLiD^TM^ NGS protocol which amplifies DNA fragments on magnetic beads for next-generation sequencing. The outcome was not satisfactory due to significant loss of sequence information and inevitable requirement of large amount of DNA input.

This issue was addressed by elegant designs using forked/Y adaptor (Illumina, US 20100273662 A1) and loop adaptor (NEB, US 20120244525 A1, WO 2012012037 A1) for Illumina next-generation sequencing. However, the procedures remain tedious and NGS-oriented commercialization has made these strategies cost-ineffective. Furthermore, the ligation and amplification efficiencies also need to be further improved for the amplification of low abundance or low copy number DNA molecules. This is particularly important because large amounts of clinical samples are examined on daily basis and significant portion of those are either of low quantity and/or low quality due to various reasons, including paraffin-embedding, long or improper storage, or at small volume or low concentration. As such, an efficient and nonselective method for full-length DNA amplification is desired.

Here, we present a robust but cost-effective single PCR primer-based full-length DNA amplification strategy, called T oligo-primed polymerase chain reaction (TOP-PCR), designed for efficient nonselective amplification of minute linear DNA fragments. Concordant with modern NGS molecular cloning strategies, which normally add an extra ‘A’ to the 3′ termini of the target DNA, we adopt a homogeneous double-stranded half adaptor (HA) which is formed by annealing ‘P oligo’ and ‘T oligo’ at room temperature. P oligo carries a phosphate group (required by ligase enzyme when later ligating HA to target DNA) at the 5′ end, while T oligo carries an extra T at the 3′ end for sticky-end ligation to the target DNA fragments. Originally the HA structure was designed for multiplex barcoded Paired-End Ditag (mbPED) sequencing library construction (US 8481699 and US 8829172). This design prevents adaptor self-ligation and allows us to maximize the ligation efficiency to an unprecedented level. Besides the protruding T-tail in one of the 3′ ends, there is another gcgc-tail at the other 3′ end. With a kinase treat followed by a ligation reaction, the extra gcgc short stretch would promote another sticky-end ligation to circularize the molecule for MmeI digestion, which would in turn generate paired-end ditags for genome-wide analysis of gene expression and regulation. Here, without the second ligation, we employed the HA strategy for efficient amplification of low abundance DNA fragments in body fluids. TOP-PCR aims to amplify, in full-length, trace amounts of linear DNA which may be present in body fluids, so that the amplified DNA fragments can be used either directly for diagnosis without DNA sequencing, or, alternatively, subjected to next-generation sequencing using customer primer or primers provided by the sequencer manufacturer.

Body fluids such as blood plasma, saliva, urine, vaginal discharge and semen, all of which may contain trace amounts of cell-free circulating DNA (cfDNA), are increasingly recognized as important liquid biopsies for non-invasive diagnostics of infectious agents, genetic defects and various diseases, including cancer^1^. Plasma cfDNA fragments derive from both mitochondrial DNA (mtDNA) and nuclear DNA released from either apoptosis or necrosis of both normal and diseased cells. Sometimes, it may also carry DNA from pathogenic microbes^2^,^3^,^4^. The concentration of plasma cfDNA has been known to be higher in cancer patients and also increases along with cancer progression - with a mean value of ~13 ng per ml of blood for normal individuals and ~180 ng per ml of blood for some metastatic cancer patients^2^, and the majority are small linear DNA fragments of a few hundred base pairs in size and with significant variation in concentration among cancer patients. Since prolonged storage and repetitive freeze-thaw cycles of whole plasma can cause substantial degradation of plasma DNA^5^,^6^, we first tested the efficiency of TOP-PCR with plasma DNA samples, which had been stored under prolonged or unfavorable conditions or isolated with inefficient method, to rescue the sequence information.

Similar to blood plasma, saliva and urine are among the most accessible body fluids. Saliva consists of a large spectrum of informative materials, including DNA, mRNA, and proteins of immunological, toxicological, hormonal and therapeutic value^7^,^8^. Applications of salivary fluid cover not only the genomics (including cancer genomics), transcriptomic and proteomics of the individual, but also the microbiota in the oral cavity. Reports have demonstrated the feasibility of using saliva in oral cancer diagnosis^9^, and the study of EGFR mutation for lung cancer patients^10^. Urine has also been reported to be useful for the study of cancer, such as colorectal and prostate cancers^11^,^12^, and the *BRAF* V600e mutations in Erdheim-Chester syndrome^13^. The cell-free DNA fragments in urine are known to be between 150–250 bp in size^14^,^15^. However, high false negative rate was reported due to limited volume or lack of advanced technology^16^.

To date, most reports are associated with diseases^17^. Here, we demonstrate the robustness and, in some cases, the essentiality, of TOP-PCR to facilitate the DNA quantity and quality assessment, as well as to improve sequence data analysis for healthy and diseased body fluid cfDNA samples. Our results also suggest that TOP-PCR can significantly enhance the completeness and the sensitivity of next-generation sequencing.

## Results

### T oligo-primed polymerase chain reaction (TOP-PCR)

**Figure 1:**
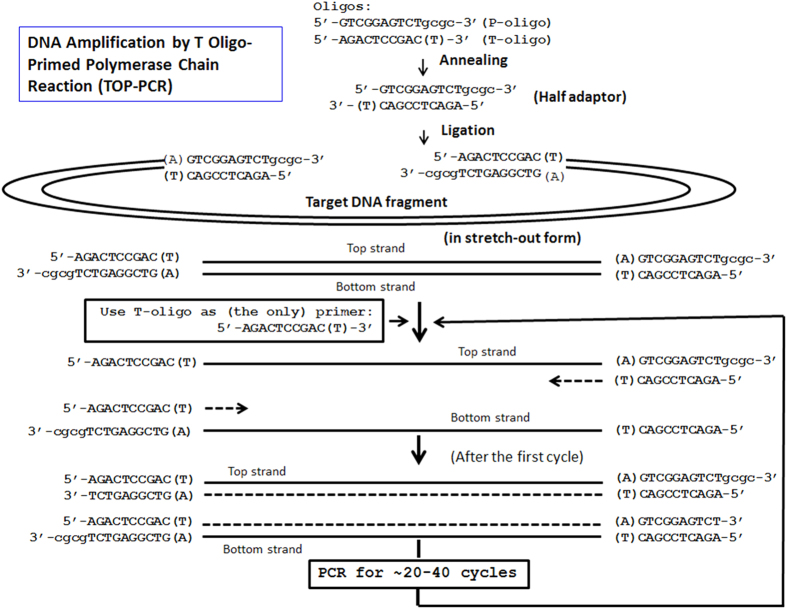
Procedure of T oligo-primed polymerase chain reaction (TOP-PCR). Half adaptor is generated by annealing P and T oligos. P oligo carries a 5′ phosphate required for ligation to target DNA fragments, while T oligo does not. During amplification, only the T oligo serves as the PCR primer. Number of amplification cycle depends on the initial concentration of the target DNA molecules and the desired final quantity.

The HA consists of a double-stranded backbone of 10 bp in length, plus a ‘T’ tail on one side and a ‘gcgc’ tail on the other (Fig. 1). Ligation of HA to both termini of the target DNA fragment defines the basic template structure for TOP-PCR amplification. This design ensures an unprecedented ligation efficiency for a strong potential to amplify low abundance and low copy number DNA fragments in the body fluids.

### Amplification of homogeneous DNA fragments

To learn the basic behavior of TOP-PCR before we practically apply the technology in biological samples, we first characterized TOP-PCR with 166 bp TBC1D1 DNA fragments made of a uniform sequence and then with PhiX DNA sample, which is composed of diverse sequences/lengths and used by Illumina as a sequencing control.

**Figure 2:**
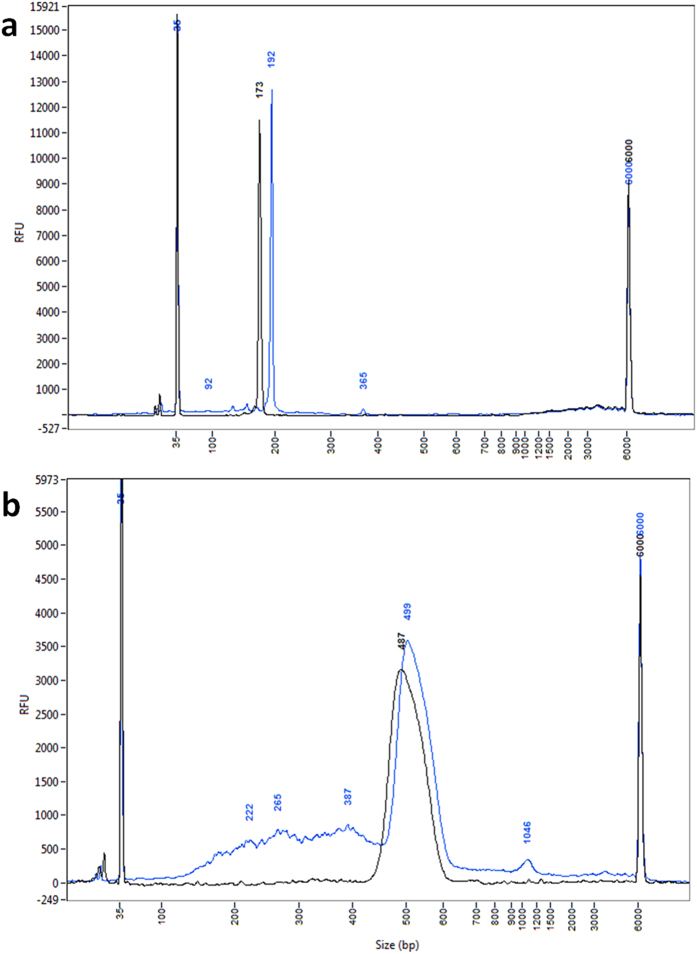
Testing of TOP-PCR on 166 bp fragment and PhiX DNA sample. (**a**) Size distribution of the original and TOP-PCR amplified 166-bp TBC1D1 DNA fragment profiled by Fragment Analyzer. (Black, original 166-bp fragment sample, 1 ng loaded; blue, after 20-cycle TOP PCR amplification of 1 ng of the original, 1 ng loaded to the FA device). The peak of the original TBC1D1 fragment should be 166 bp in size, but is shown as a 173 bp peak due to the imprecision of the device. (**b**) Size distribution of the original and TOP-PCR amplified PhiX DNA fragments profiled by FA. (Black, original PhiX control DNA, 2.8 ng loaded; blue, after 20-cycle TOP-PCR amplification of 1 ng of the original, 4.6 ng loaded). Size markers: 35 bp and 6000 bp peaks. Size of two HAs added to each DNA fragment: ~22 bp.

With a total length of 166 bp, the TBC1D1 DNA fragment was designed to mimic the mono-nucleosomal DNA fragments. As expected, addition of two HAs resulted in a distinct increase in size and TOP-PCR was able to well maintain its size profile (Fig. 2a). Due to the imprecision of the Fragment Analyzer, the original fragment is shown in the figure as the peak of 173 bp, while the amplified fragment is shown as the peak of 192 bp (188 bp, or 166 + 22, is expected). Such bias in size estimation is common for such types of devices and will be shown in all related figures throughout the report. PhiX DNA sample contains DNA fragments of variable sequences and sizes. TOP-PCR amplified all DNA species (Fig. 2b), including DNA of the major peak together with those of either smaller or larger sizes. A distinct peak shift was also observed.

### TOP-PCR amplification of plasma cfDNA

**Figure 3:**
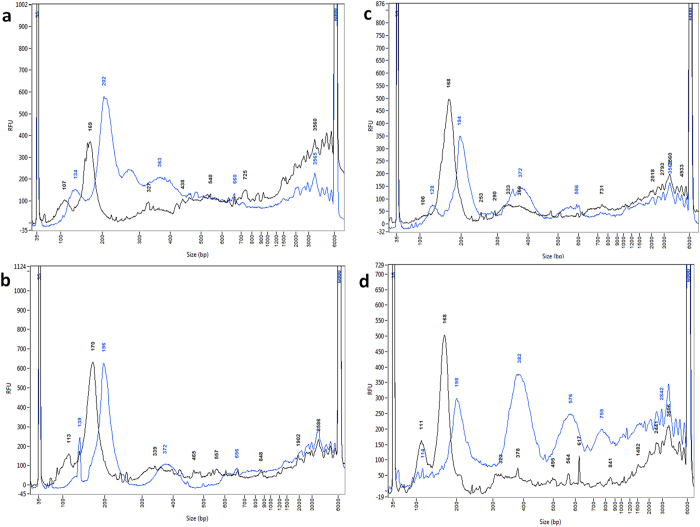
Size profile comparison between the original and the TOP-PCR amplified normal plasma DNA samples. Plasma DNA samples (1 ng each) from four normal individuals, including two males (YFH and THC, shown in (**a**,**b**), respectively) and two females (EJC and BBC, shown in (**c**,**d**), respectively), were each amplified by TOP-PCR for 20 cycles and then displayed in parallel with the originals by Fragment Analyzer. Black, original, 1 ng loaded; blue, after TOP-PCR, 1 ng loaded. Size markers: 35 bp and 6000 bp peaks. Size of two HAs added to each DNA fragment: ~22 bp.

We then characterized TOP-PCR using samples isolated from the plasmas of a cancer patient and healthy individuals, as well as saliva and urine samples from healthy individuals. The plasma DNA samples were prepared and each amplified by TOP-PCR. Again, results clearly demonstrated that TOP-PCR could maintain the profiles (Fig. 3). Back in 2001, Jahr, S. *et al*. demonstrated that, together with large DNA fragments which may be generated from necrosis, plasma samples of cancer patients carry DNA fragments of various nucleosomal sizes and these DNA fragments are potentially resulted from apoptosis of both healthy and diseased cells^2^. We have noticed that, besides the mono-nucleosomal fragments, TOP-PCR also emphasized, at least, the di-nucleosomal (Fig. 3a–d) and, in some cases, tri-nucleosomal (Fig. 3c,d) and above (Fig. 3d). The underlying reason remains unclear. However, since these pools may comprise DNA fragments derived from both normal and diseased cells, TOP-PCR imposes no selection upon either origin.

### TOP-PCR amplification of saliva and urine cfDNA

**Figure 4:**
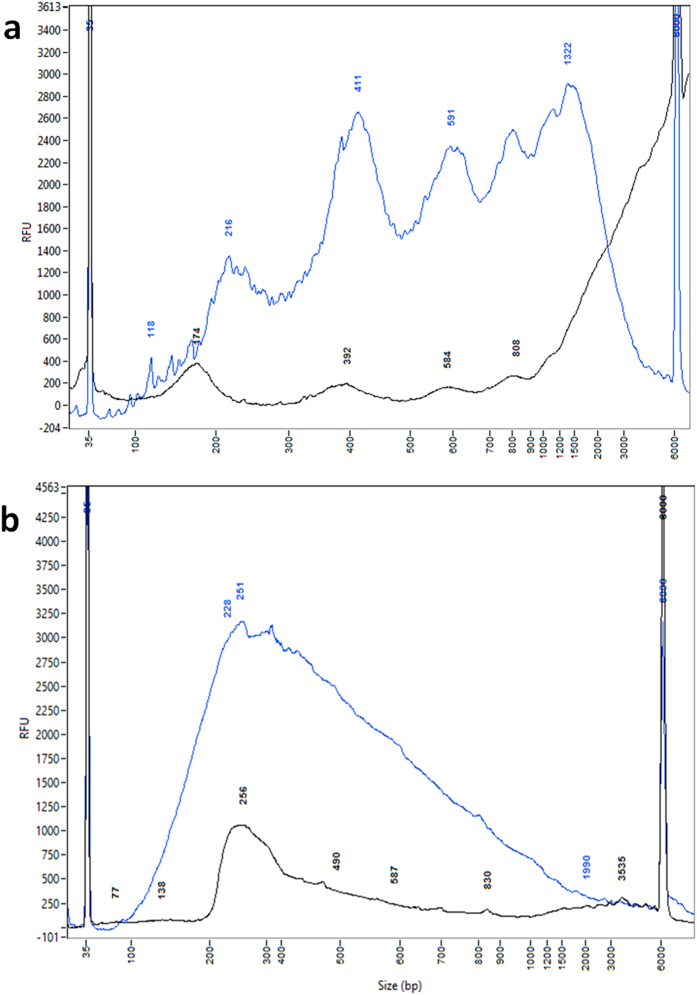
TOP-PCR amplification of saliva and urine cfDNA. (**a**) Size profile comparison between the original and TOP-PCR amplified normal saliva DNA samples. The saliva cfDNA from a healthy male individual (YFH) was amplified by TOP-PCR and displayed in parallel with the original. (Black, 5 ng of original; blue, 1 ng of TOP-PCR product). (**b**) Comparison between the original and TOP-PCR amplified normal urine cfDNA samples. Urine sample from the same healthy male (**a**) was tested. (Black, original urine DNA; blue, 40-cycle TOP-PCR amplification of 0.1 ng of the original. 5 ng each was displayed by Fragment Analyzer. Size markers: 35 bp and 6000 bp peaks. Size of two HAs added to each DNA fragment: ~22 bp.

The original human saliva DNA showed a pattern very similar to that of plasma DNA, but with significantly larger amount of large-sized DNA (Fig. 4a). Again, TOP-PCR was able to maintain the size profile while at the same time enhancing the resolution of nucleosomal-sized DNA fragments. Notice that the saliva DNA contains significant amount of large-sized chromosomal DNA presumably derived from necrosis, while under the current condition setting, the maximal size can be faithfully amplified by TOP-PCR is about 1.5 Kb, still within the size range (180–1,000 bp) for apoptotic DNA fragments^17^. If the large-sized DNA population is of interest, one can isolate it by agarose gel electrophoresis prior to sequencing.

Interestingly, TOP-PCR amplification of urine cfDNA revealed a distinct profile which is very different from that of plasma and saliva DNA samples, i.e., nucleosomal fragment DNA profile was not observed (Fig. 4b). This is partially due to the fact that the glomerular filtration apparatus does not permit the passage of nucleosomal DNA fragments to pass through.

### Reproducibility of TOP-PCR

**Figure 5:**
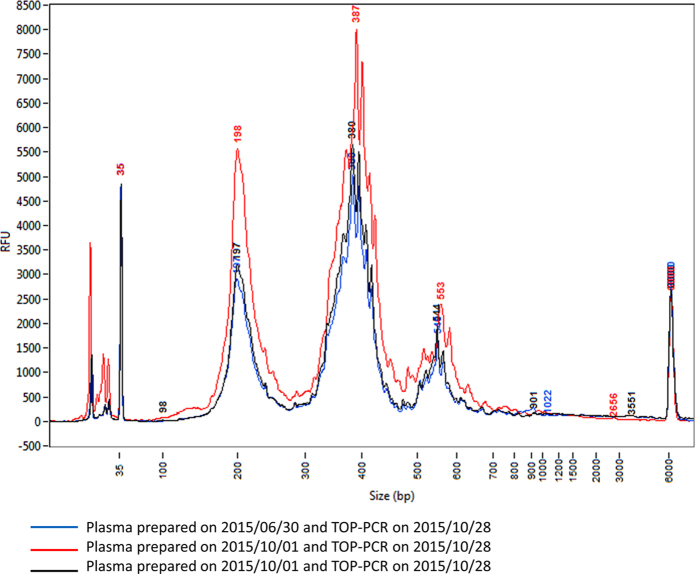
Test of TOP-PCR reproducibility and cfDNA consistency. Two blood samples were separately collected from a healthy male (**YFH**) on June 30, 2015 and October 28, 2015. Plasmas were prepared right after the blood collections and stored at −80 °C. Samples of cfDNA were extracted right before TOP-PCR reactions conducted on October 28, 2015. Blue, plasma stock prepared on June 30, 2015; red and black, plasma stock prepared on October 1, 2015. Size markers: 35 bp and 6000 bp peaks. Size of two HAs added to each DNA fragment: ~22 bp.

To validate the reproducibility of TOP-PCR, we isolated plasma DNA from a healthy male (YFH) and then repetitively preformed TOP-PCR on the same sample for a few times. Results indicated that TOP-PCR is highly reproducible (Fig. 5).

### Comparison between TOP-PCR and Illumina’s library construction using loop adaptor

It would be interesting to compare TOP-PCR to Illumina’s amplification method. Here, TOP-PCR (using half adaptor) was compared to the Illumina PCR amplification procedure using loop adaptor, which was randomly chosen, and the comparison was conducted for both low (with unknown concentration) and high (20 ng) initial DNA inputs.

### TOP-PCR vs. Illumina’s protocol at low DNA concentration

**Figure 6:**
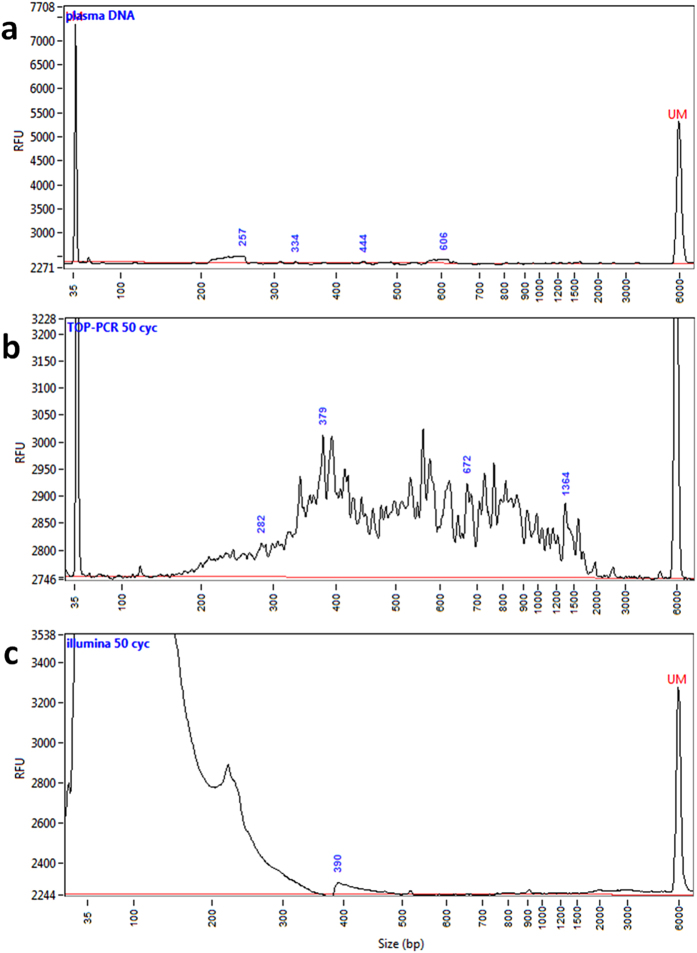
Comparison of TOP-PCR with Illumina’s PCR method using low amount of DNA as the input. (**a**) One micro-liter of original ovarian cancer plasma cfDNA sample with unknown concentration. (**b**) Same DNA sample but after 50 cycles of amplification by TOP-PCR. (**c**) Same DNA sample but after 50 cycles of amplification using Illumina’s protocol. Size markers: 35 bp and 6000 bp peaks. Size of two HAs added to each DNA fragment: ~22 bp.

As shown in Fig. 6, TOP-PCR was able to rescue the DNA fragments from a sample with a DNA concentration far below the undetectable level by regular devices. On the other hand, Illumina’s method generated significant amount of adaptor dimers instead. PCR products were detected by Fragment Analyzer (FA) after 30 cycles. Here, we performed PCR for 50 cycles to investigate further details.

Further analysis on the sequence contents in the amplified profiles indicated that there is a dramatic difference between the amplified products by TOP-PCR and that by Illumina’s procedure (Table 1). Here, we isolated the DNA fragments by gel excision from the PCR products amplified by Illumina’s PCR and TOP-PCR methods, cloned the isolated DNA into pZBack vector (Tools Biotechnology), used the vectors to transform E. coli, cultured 20 colonies for each, made mini-preps and sequenced the inserts with Sanger sequencing. Clones with empty inserts and inserts with adaptor dimers or polymers were removed. The remaining sequences were mapped against nr/nt database (NCBI) to identify the origins of sequences –Among the 20 pZBack clones reported for Illumina’s method, none mapped to the human genome. On the other hand, among the 20 pZBack clones reported for TOP-PCR method, 8 mapped to the human genome, indicating the superiority of TOP-PCR over Illumina’s method and its robustness in cloning trace DNA molecules that may be present in plasma and, in theory, in other body fluids as well.

### TOP-PCR vs. Illumina’s protocol using high DNA input

**Figure 7:**
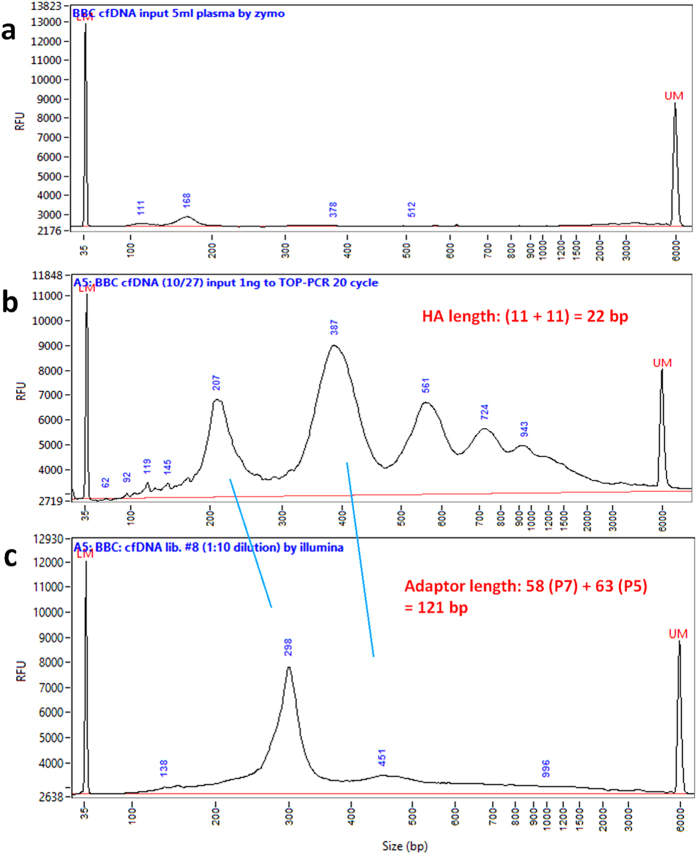
Comparison of TOP-PCT with Illumina’s PCR method using 20 ng of DNA. (**a**) One nano-gram original plasma cfDNA sample isolated from a healthy female (BBC). (**b**) Same DNA sample but after 20 cycles of amplification using TOP-PCR. (**c**) Same DNA sample but after 20 cycles of amplification using Illumina’s protocol. Size markers: 35 bp and 6000 bp peaks. Size of two HAs added to each DNA fragment: ~22 bp.

**Figure 8:**
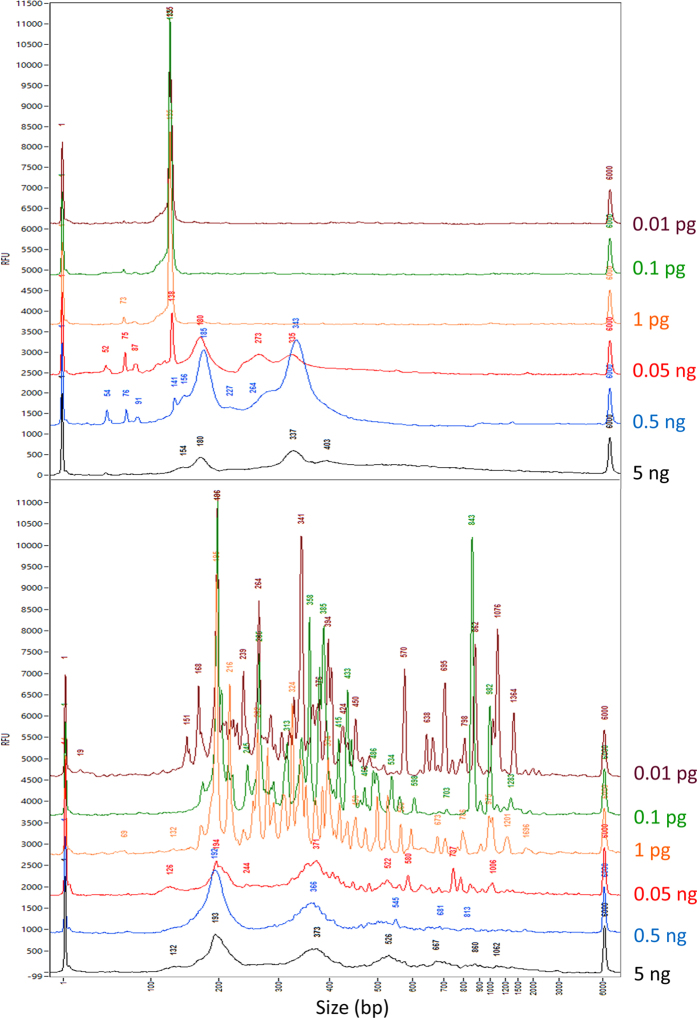
Comparison of TOP-PCR to Illumina’s PCR method using serial dilutions of plasma cfDNA sample. Serial dilutions (5 ng–0.01 pg) of a plasma cfDNA sample isolated from a healthy female (**BBC**) was prepared and the cfDNA is amplified using either Illumina’s PCR or TOP-PCR. **TOP panel:** profiles generated by Illumina’s PCR method. **Lower panel:** profiles generated by TOP-PCR. Notice that the RFU values are no longer accurate because of figure overlay. PCR cycle numbers: 30 for 5–0.5 ng; 40 for 0.05 ng–0.01 pg. Size markers: 1 bp and 6000 bp peaks. Size of two HAs added to each DNA fragment: ~22 bp.

Comparison with high DNA input revealed that DNA amplification by Illumina’s method mainly focused on the mono-nucleosomal fragments, while TOP-PCR broadly amplified both mono- and multiple nucleosomal DNA fragments (Fig. 7). This phenomenon is consistently observed (also see Fig. 8 shown below). We suspect the difference may result from the following facts: (1) HA is shorter than loop and Y adaptors; (2) HA is linear and without secondary structure, while loop and Y adaptors have secondary structures; and (3) TOP-PCR uses only a single oligo as PCR primer. Short and linear adaptor is supposed to be more accessible for target DNA fragments during ligation, while a short oligo is also more accessible for PCR priming. More study is needed to further understand the reasons that cause the difference.

### Comparison of TOP-PCR to Illumina PCR using serial dilutions of a plasma cfDNA sample

To further understand the phenomena shown above (Figs 6 and 7), we compared these two methods using serial dilutions of a plasma DNA sample. Again, PCR on serial dilutions of the same plasma DNA sample isolated from a healthy female (BBC) revealed dramatic differences in amplification patterns between TOP-PCR and Illumina’s PCR method (Fig. 8). Results also indicated that Illumina’s method starts making adaptor dimers when 0.05 ng (50 pg) cfDNA was used as the input and became dysfunctional when input DNA is below this level. On the other hand, TOP-PCR maintained the full range of profile during amplification even if the input DNA reached 0.01 pg.

### Comparison of our procedure using HA against Illumina’s procedure using Loop and Y adaptors

Both experimental procedure and adaptor play a key role in experimental efficacy. An effective experimental procedure should be short and comprises a minimal number of, but well optimized, experimental steps; but at the same time, the retention of materials, especially for low abundance and low copy number genetic materials, is maximized.

To further understand the differences in efficiencies of experimental procedure, adaptor ligation, and PCR amplification between HA, loop adaptor and Y adaptor, we conducted a number of experimental replicates using these adaptors together with their corresponding procedures. Results clearly demonstrated that HA procedure outperforms the other adaptors and their corresponding procedures (Table 2).

DNA recovery rate indicates whether DNA isolation (with magnetic beads) is required by the protocol. If required, 20% loss is expected, as estimated by the beads provider and experiences. As such, 100% indicates it is not required, while 80% indicates it is required. Ligation efficiency is an estimation based on the results shown in Fragment Analyzer (F.A.), which displays un-ligated, single-side ligated, and double-side ligated target DNA fragments in separate peak regions so that an estimation for each fraction is feasible. Previously we had tested HA for a number of times with ligation time ranging from 30 min to overnight. As expected, results indicated that both ligation efficiencies of single-sided and double-sided ligations increased along with ligation time. However, we have to balance between the ligation time, which should be kept within a few hours, and ligation efficiency, which should be maintained at a high level. Thus, we decided to choose 4 hr as the ligation time and this is most likely to be used in the future. Notice that the procedures used for Illumina sequencing is also much more tedious than our procedure.

## Discussion

Initially, TOP-PCR was invented to amplify trace amounts of DNA fragments in body fluids for the study and diagnosis of diseases (such as cancer), bacterial/viral infections and prenatal genetic defects, using either conventional diagnostic approaches or next-generation sequencing. To make it easy to use, optimization of experimental conditions has been conducted. The whole procedure is composed of three major steps: end repair/A-tailing, ligation of HA to target DNA and TOP-PCR amplification. We optimized the experimental conditions for each step and evaluated the efficiency. Currently, no DNA purification is needed between these steps, and at the same time the amplification efficiency is maintained at an unprecedented level. In fact, to the best of our knowledge, this is the most efficient and the simplest experimental procedure of this kind.

HA is a small adaptor originally designed for cloning of barcoded paired-end ditags (bPED) and, later, for amplification of minute amount of cfDNA due to its superior ligation efficiency and flexible ligation time, which can be extended to overnight or further. The HA was so designed because each terminus of a target DNA fragment can ligate only to one HA, even at excess HA concentration or elongated incubation time. Thus, after circularization, only one full adaptor can be generated. These features cannot be achieved by directly using full adaptor for bPED cloning. We took advantage of the design and applied it to TOP-PCR for efficient DNA amplification.

Compared to HA, Y and Loop adaptors are much larger and were originally designed for sequencing, not for DNA amplification. Their designs are elegant for sequencing, but not for amplification. In fact, there are significant drawbacks for adaptors and procedures used by Illumina. Besides that shown in Table 2, their protocols are much more tedious and require large amounts of starting material for making a sequencing library (500 pg–1 μg as shown in page 4 of NEBNext UltraII DNA Library Prep Kit for Illumina, or >100–200 ng input gDNA as shown in page 5 of TruSeq Nano DNA Library Prep Reference Guide). With TOP-PCR, one just needs a small fraction of theirs and it is thus suitable for cfDNA analysis for liquid biopsies.

In summary, our method conveys a number of advantages. Firstly, because the HA is a short homogeneous adaptor, it is able to ligate efficiently to the termini of DNA fragments. As such, most DNA fragments can be ligated and subjected to amplification. Secondly, the range of insert (target DNA) size well matches the sizes of cfDNA fragments in the body fluids, making it suitable for body fluid DNA amplification and sequencing. Thirdly, HA molecules do not self-ligate so that the concentration can be well maintained. Fourthly, TOP-PCR utilizes only one PCR primer and this primer was previously used to make the adaptor, making it simple and cost-effective. Fifthly, once amplified, DNA samples can be directly subjected to next-generation sequencing using customer primer. Moreover, we have also generated a protocol which allows us to perform end-repair/A-tailing, adaptor ligation and TOP-PCR in a single tube, without DNA purification until PCR has been conducted. This single-tube procedure allows us to maximize the retention of DNA molecules throughout the experiment so that the overall efficiency can be increased significantly.

Our results also suggest a potential application of TOP-PCR for the detection of apoptotic cell death. As demonstrated with plasma, saliva and urine samples, by coupling with simple devices TOP-PCR was able to distinguish DNA fragments of various nucleosomal sizes resulted from apoptotic cell death, making it readily distinguishable from that resulted from necrotic cell death or any other means. As such, by using TOP-PCR method one can bypass the conventional staining methods using multiple reagents in tedious experimental procedures.

Overall, our results demonstrate the robustness of TOP-PCR in amplifying minute DNA fragments for diagnosis. By coupling TOP-PCR with next-generation sequencing, we will be able to significantly improve the sensitivity of sequence information in body fluids.

## Methods

### Preparation of 166 bp DNA fragment and PhiX DNA

To test TOP-PCR on uniform DNA fragments, a region of 166 bp in *TBC1D1* gene (seq.: TGATCCAAAACAGAAAAACAGTGATAACTGTTTTGCTGAGTTCCCAGACCCTTCCCAAGATGGAACCAATAACATTCACAGCAAGGAAACATCTGCTTTCTAACGAGGTCTCGGTGGATTTTGGCCTGCAGCTGGTGGGCTCCCTGCCTGTGCATTCCCTGACCAC) was amplified with conventional PCR (primer sites underlined). The size (166 bp) was chosen to mimic the size of mono-nucleosomal DNA fragments previously identified in blood plasma^2^. The PhiX DNA is the same PhiX Control v3 DNA (Illumina, Inc. #FC-110-3001) used by Illumina as a control for sequencing.

### Cell-free DNA isolation from blood plasma and saliva

Fresh blood samples were collected into commercially available EDTA anticoagulant-treated tubes and mixed well with the anticoagulation and centrifuged at 3,000 × g and 4 °C for 15 min (Eppendorf, Centrifuge 5810 R). The upper light yellow phase was transferred into a new tube and centrifuged for another 10 min at 1,000–2,000 × g to completely remove cells and platelets. The plasma samples can be stored at −80 °C. Plasma DNA was isolated by Quick-cfDNA Serum and Plasma Kit (Zymo Research, D4076) following the manufacturer’s instructions. Briefly, plasma samples (<5 ml each) were incubated with proteinase K in digestion buffer for 30 min in a 55 °C water bath, then mixed with DNA binding buffer and loaded into the column to allow DNA to bind to the column membrane. After wash, cfDNA was eluted into 35 μL ddH_2_O, and quantified by Qubit 2.0 Fluorometer (Invitrogen) or Fragment Analyzer^TM^ (AATI).

Saliva cfDNA was also isolated by using the same Quick-cfDNA Serum and Plasma Kit. About 1 mL of saliva was used in each preparation.

### Cell-free DNA isolation from urine

Urinary cfDNA was isolated using QIAamp Circulating Nucleic Acid Kit (QIAGEN, 55114) following the manufacturer’s instructions. Briefly, urine samples were mixed with proteinase K, buffer ACL and buffer ATL, and incubated in a 60 °C water bath for 30 min. ACB binding buffer was added to the mixture and the preparation was then loaded into the mini-column. Then, the liquid was flushed off by vacuum pump and the DNA was eluted into ddH_2_O and quantified by Qubit 2.0 and/or Fragment Analyzer.

### Preparation of half adaptor (HA)

Ligation of half adaptor to the target DNA fragments is essential for TOP-PCR. The HA was prepared by annealing P oligo (5′-pGTCGGAGTCTgcgc-3′) and T oligo (5′-AGA CTC CGA Ct-3′) at room temperature at 1:1 molar ratio. The P oligo carries a phosphate at the 5′ end and an additional ‘gcgc’ sequence previously designed for another sticky-end ligation to make paired-end ditag libraries. Here the ‘gcgc’ tail is not required for TOP-PCR, but we still keep it for potential use in the future. The T oligo has an extra ‘T’ at the 3′ end but has no 5′ phosphate.

### Ligation of HA to DNA fragments

Prior to ligation, cfDNA fragments were end-repaired and 3′ A-tailed using NEBNext Ultra End Repair/dA-Tailing Module (NEB, E7442S/L). The ligation mixture (30 μL) contains end repaired/A-tailed cfDNA (normally ranging between 1 pg to a few ng), HA (at 50:1 ratio against cfDNA), 1X ligation buffer, and 1uL NEB Quick Ligation^TM^ Kit (NEB, M2200L). The reaction was incubated at 25 °C overnight in a thermocycler. The ligation mixture can be directly subjected to TOP-PCR amplification with or without purification. An at least 4X dilution is needed, if without purification.

### TOP-PCR

TOP-PCR employs T oligo as the only PCR primer to amplify DNA fragments. The reaction mixture (50 μL) contains 10 μL DNA template (in desired quantity), 0.64 μM T oligo, 1.5 mM MgSO_4_, 0.2 mM dNTPs, 1 X buffer, and 1unit Novagen KOD hot start DNA polymerase (EMD Millipore Co., 71086-3). The reaction conditions: initial denaturation at 94 °C for 3 min; (denaturation at 94 °C for 30 sec; annealing at 27 °C for 30 sec; elongation at 65 °C for 1 min) for 20–40 cycles; final elongation at 65 °C for 5 min; then hold at 4 °C. PCR products were purified with AMpure beads. When sequencing is desired, TOP-PCR products can be directly ligated to sequencing adaptors without fragmentation. Alternatively, the amplified products can be sonicated (e.g., by S2/E210 Focused-ultrasonicator (Covaris) prior to ligation.

### Comparison of TOP-PCR with Illumina’s method for DNA with unknown quantity

For TOP-PCR amplification, 1 μL plasma DNA (with unknown quantity) from an ovarian cancer patient was end-repaired/3′ A-tailed following the instructions provided by the manufacturer (NEB, E7442S/L). Briefly, the reaction mixture (25 μL) contained plasma DNA, 1X NEBNext end repair reaction buffer and 1 μL NEBNext end repair enzyme mix, and the mixture was incubated at 25 °C for 20 min followed by 72 °C for 20 min in a thermocycler. The plasma DNA reparation was ligated to HA in a 30 μL reaction mixture under the same conditions mentioned above. TOP-PCR reaction also followed the procedure mentioned above.

For Illumina sequencing library construction, 1 μL plasma DNA (with unknown quantity) from an ovarian cancer patient was mixed in 1X end repair/A-tailing reaction buffer with end prep enzyme mix to a final volume of 65 μL and then incubated at 20 °C for 30 min followed by another 30 min in a 65 °C thermocycler, as instructed by the manufacturer (NEBNext, E7442S/L). For adaptor ligation, the DNA preparation was mixed with 15 μL Blunt/TA ligase master mix, 2.5 μL 1:10 diluted (based on the manufacturer’s instructions) NEBNext adaptor (1.5 μM final), and 1 μL ligation enhancer to a final volume of 83.5 μL. The mixture was incubated at 20 °C for 15 min in a thermocycler. Then, 3 μL USER™ enzyme, used to cut open the loop, was added to the ligation mixture and the preparation was incubated at 37 °C for another 15 min.

After ligation, free adaptors were removed and cleaned up by AMpure beads and DNA was collected in 23 μL nuclease free water for PCR amplification. As instructed by the manufacturer, the PCR reaction mixture (50 μL) contained 25 μL NEBNext high fidelity 2X PCR master mix (final 1X), 1 μL index primer, and 1 μL universal PCP primer and ran PCR under the following conditions: initial denaturation at 98 °C for 30 sec; (denaturation at 98 °C for 10 sec, annealing at 65 °C for 30 sec, extension at 72 °C for 30 sec) for 15 cycles or more (when compared with TOP-PCR); final extension at 72 °C for 5 min, then hold at 4 °C.

Free primers were removed by AMpure beads and DNA was collected in 25 μL nuclease free water. 2 μL of collection was assessed by Fragment Analyzer, and part of the other 23 μL was used for in cloning and transformation. 20 colonies were validated by Sanger sequencing.

### Comparison of TOP-PCR with Illumina’s method for high concentration of target DNA

Normal female plasma DNA was first quantified by Qubit Fluorometer. We then applied 20 ng to be amplified by Illumina’s method for 20 cycles following the instructions provided by Illumina for input quantity ranging between 5 ng–1 μg. All the reagents were also provided by Illumina. In parallel, we applied another 1 ng to be amplified by TOP-PCR for 20 cycles. All the procedures and conditions (including end-repair/A-tailing, adaptor ligation, DNA purification, TOP-PCR mix preparation and conditions, and post TOP-PCR DNA purification) are exactly the same as described above.

### Ethical Approval and Consent

All methods were performed in accordance with relevant guidelines and regulations.

All experimental protocols were approved by IRB on Biomedical Science Research/IRB-BM, Academia Sinica (AS-IRB03-105025), issued to Taiwan Biosignature Project for Breast Cancer: Investigate the Presence of Circulating DNA with Specific Mutations Identified by Whole Exome Sequencing of Early Onset Breast Cancer.

Informed consent was also obtained from all subjects.

## Additional Information

**How to cite this article**: Nai, Y.-S. *et al*. T Oligo-Primed Polymerase Chain Reaction (TOP-PCR): A Robust Method for the Amplification of Minute DNA Fragments in Body Fluids. *Sci. Rep.* **7**, 40767; doi: 10.1038/srep40767 (2017).

**Publisher's note:** Springer Nature remains neutral with regard to jurisdictional claims in published maps and institutional affiliations.

## Figures and Tables

**Table 1:** Sequence analysis of TOP-PCR and Illumina PCR products amplified from equal amount of ovarian cancer plasma cfDNA with undetectable quantity.

PCR Method	#/insert within pZBack flanking seq.	#Seq.	Insert within adaptors
Illumina	2 had no reports (from Sanger sequencing)		
	8 with loop adaptor seq.	0	*H. sapiens*
		2	*E. coli*
		6	unknown
	5 empty (with pZBack flanking seq. only)		
	5 unknown sequences		
TOP-PCR	13 with HA seq.	8	*H. sapiens* seq.
		3	*E. coli* seq.
		1	*Aeromonas veronii* (a bacterium) seq.
		1	unknown seq.
	6 empty (with pZBack flanking seq. only)		
	1 unknown sequence		

**Table 2:** Comparison between HA, loop adaptor and Y adaptor on experimental procedure and ligation efficiency.

Adaptor	Input DNA (ng)	Ligation time	DNA recovery rate (%)		Ligation efficiency (%)			DNA recovery rate (%)	Total efficiency (%)	Ave. (%)
			After end-repair	After A-tailing	Un- ligated	ss- ligated	ds- ligated	After ligation		
HA	10	4 hr	100	100	13.9	20.1	66.0	80	52.8	52.5
	10	4 hr	100	100	15.1	22.3	62.6	80	50.1	
	10	4 hr	100	100	10.5	21.3	68.2	80	54.6	
Loop	100	15 min	100	100	35.0	38.6	26.4	80	21.1	22.2
	100	15 min	100	100	36.4	34.6	29.0	80	23.2	
Y	100	30 min	80	100	3.1	28.4	68.5	80	43.8	44.5
	100	30 min	80	100	3.3	26.0	70.7	80	45.2	

100% DNA recovery rate indicates that DNA isolation (with beads) is not required, and 80% indicates that it is required. Ligation efficiency is an estimation based on the results shown in Fragment Analyzer, which displays un-ligated, single-side ligated, and double-side ligated target DNA fragments in different peak regions.
